# Drug Delivery by Ultrasound-Responsive Nanocarriers for Cancer Treatment

**DOI:** 10.3390/pharmaceutics13081135

**Published:** 2021-07-26

**Authors:** Kristin Entzian, Achim Aigner

**Affiliations:** 1 Faculty of Medicine, Rudolf-Boehm-Institute for Pharmacology and Toxicology, Clinical Pharmacology, Leipzig University, 04107 Leipzig, Germany; kristin.entzian@bioserv.de; 2 BIOSERV Analytik und Medizinprodukte GmbH, 18059 Rostock, Germany

**Keywords:** nanocarrier, ultrasound, tumor therapy, nanodrugs, ultrasound-triggered drug delivery

## Abstract

Conventional cancer chemotherapies often exhibit insufficient therapeutic outcomes and dose-limiting toxicity. Therefore, there is a need for novel therapeutics and formulations with higher efficacy, improved safety, and more favorable toxicological profiles. This has promoted the development of nanomedicines, including systems for drug delivery, but also for imaging and diagnostics. Nanoparticles loaded with drugs can be designed to overcome several biological barriers to improving efficiency and reducing toxicity. In addition, stimuli-responsive nanocarriers are able to release their payload on demand at the tumor tissue site, preventing premature drug loss. This review focuses on ultrasound-triggered drug delivery by nanocarriers as a versatile, cost-efficient, non-invasive technique for improving tissue specificity and tissue penetration, and for achieving high drug concentrations at their intended site of action. It highlights aspects relevant for ultrasound-mediated drug delivery, including ultrasound parameters and resulting biological effects. Then, concepts in ultrasound-mediated drug delivery are introduced and a comprehensive overview of several types of nanoparticles used for this purpose is given. This includes an in-depth compilation of the literature on the various in vivo ultrasound-responsive drug delivery systems. Finally, toxicological and safety considerations regarding ultrasound-mediated drug delivery with nanocarriers are discussed.

## 1. Introduction

Cancer is one of the leading causes of death worldwide [1] and, despite the continuous progress in modern medicine, effective tumor diagnosis and treatment remain challenging problems. Chemotherapy as an important therapeutic strategy has been widely used for cancer treatment. The often inefficient delivery of chemotherapeutic agents due to several barriers (anatomical, e.g., membranes; physiological, e.g., kidney filtration; and pathophysiological, e.g., tumor heterogeneity) as well as drug characteristic restrictions (e.g., solubility, stability) usually encountered leads to unsatisfactory anticancer efficacy with severe toxic side effects [2].

The worldwide need for therapeutics with higher efficacy and/or improved safety and toxicological profiles has promoted the rapid evolution of nanomedicines, including the development of systems for drug delivery, imaging and diagnostics in the nanoscale [3]. Nanoparticles loaded with drugs (nanocarriers) can be designed for improving efficiency and reducing toxicity and can be made of different (organic or inorganic) materials. The phenomenon known as the “enhanced permeation and retention” (EPR) effect (accumulation of macromolecules in tumor tissue due to leaky vasculature and deficient lymph system [4]) is exploited as a passive targeting technique for drug delivery, but the accumulation efficiency is often very poor [5,6,7,8].

Modifications of nanoparticles in particle size, shape, surface properties and composition can be implemented for improving their functionality and accumulation efficiency in the tumor tissue. Such modifications can facilitate drug delivery across several biological barriers as well as mediate cell penetration, solubilization, protection from degradation and renal filtration, enhancement of bioavailability, sustained release, immunoevasion (e.g., nanoparticle escape from RES by PEGylation), safe delivery of higher drug doses to tumor cells under avoiding side effects [9], targeting, and triggered activation (controlled release systems) [10,11]. In controlled release systems (smart drug delivery systems, SDDS), stimuli-responsive nanocarriers (e.g., responsive to ultrasound) are used that are able to release their payload on demand at the tumor tissue site, preventing premature drug loss.

Ultrasound (US), which has been extensively applied in clinics for both diagnostic purposes and treatment, is considered as one of the promising triggers for stimuli-responsive drug delivery nanosystems due to its capability to non-invasively penetrate deeply into the tissue without damaging it [12]. Beyond efficacy considerations, toxicological aspects (biocompatibility) and safety issues play an important role in this context as well and are major barriers for the translation of this promising technology towards clinical application [13]. Therefore, they should be already addressed in early stages of SDDS development.

The aim of this paper is to highlight successful examples of recent developments in the field of US-triggered drug delivery nanosystems for cancer treatment, including toxicological and safety considerations in this respect.

## 2. Physics of Ultrasound and Its Biological Effects

Due to the physical characteristics of US and the cost effective, safe and non-invasive manner of its use [12], US is widely applied for either diagnostic imaging or therapeutic purposes (e.g., tumor tissue ablation [14], physiotherapy [15], kidney stone comminution [16]), or the combination of both (“Theranostics”)) [17].

US consists of acoustic waves (a form of pressure waves) with a frequency above the upper limit of human hearing (>20 kHz) [14,18,19] which need a medium to travel through (unlike light or electromagnetic waves) [20,21]. Acoustic waves propagate mostly longitudinally in gases or liquid. In solids, transversal waves due to shear stress have been found to additionally occur as well [22]. In general, US waves possess physical properties, such as attenuation, reflection, refraction, amplification, absorption, and scattering, that are inherent in any wave [20,21].

**Figure 1:**
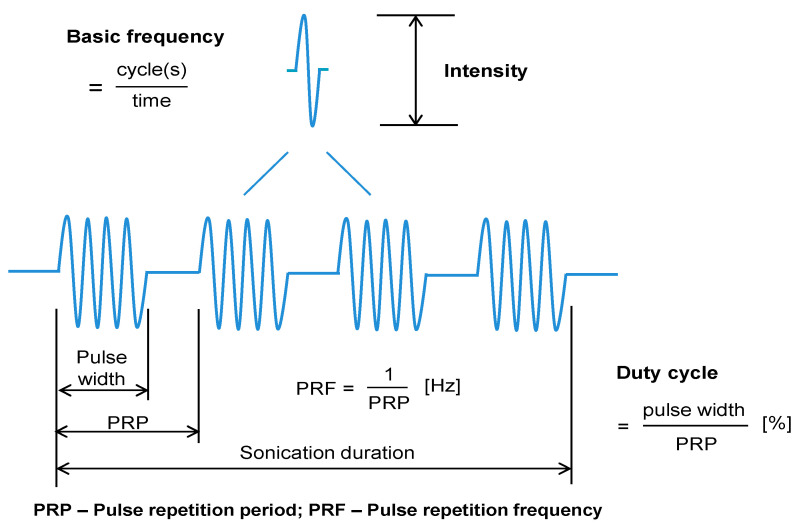
Physical US parameters (adapted from [24], Frontiers, 2020.).

The source of US is most often a transducer containing a piezoelectric crystal, which is capable of converting an electrical signal into mechanical pressure waves [19]. These pressure waves cause, when passing, local oscillatory motion of particles through the transmitting medium which results in a local density change in the medium (succession of compression and decompression events). The applied acoustic pressure (measured in Pa) is directly related to the amount of energy received by the targeted tissue [14,19]. Biological effects induced by US application can be influenced by varying different parameters such as frequency, intensity or exposure time. In addition, US can be applied in a continuous or discontinuous mode (pulsed mode). A continuous application of US for a certain period of time of tissue exposure leads to overheating and tissue damage. This effect is clinically exploited for tumor tissue ablation [23]. In cases where tissue overheating is undesired, continuous waves can be broken down into several US pulses, so that energy dissipation occurs between pulses. Pulses can vary in length and are repeated periodically at a given frequency (repetition per second), which determines the duty-cycle (DC) of the US application [14] (Figure 1).

### 2.1. Frequency

The frequency, which can be expressed as the ratio of speed and wavelength, is the most common parameter to describe US waves [25]. A wide range of US frequencies can be applied on the human body for medical applications: low frequency (20–200 kHz), medium frequency (0.7–3 MHz), and high frequency (>3 MHz) [26,27]. Frequency influences spatial resolution and tissue penetration depth: high frequency leads to high resolution but low tissue penetration, whereas low frequency leads to limited resolution with high tissue penetration. For diagnostic imaging purposes, higher frequencies (≥1 MHz) are required than for therapeutic purposes to obtain a suitable image resolution [14,19,28,29].

### 2.2. Intensity and US-Focus

The US intensity (measured in W/cm^2^) represents the amount of energy delivered to the desired location and is defined as the ratio between the amount of power carried by the acoustic wave and the surface on which it is applied. Thus, the US intensity correlates with the acoustic pressure, the density of the medium and the US propagation speed in the medium. The intensity of US can be classified into two groups: low intensity US (ranging from 0.125–3 W/cm^2^) leading to reversible tissue changes; and high intensity US (ranging from 3 to several thousand W/cm^2^), which induces hyperthermia and leads to irreversible tissue changes [14,19].

US can be provided by non-focusing transducers and focusing transducers. Non-focusing transducers are typically applied to achieve physical effects or to enhance transdermal drug delivery (sonoporation) [30]. The use of special transducers allows the generation of focused US (FUS) and, thus, the deposition of a large amount of energy on a small zone at a defined depth within the body in a non-invasive manner, without harming the surrounding tissue [31]. Low frequency ultrasound (LFUS) is difficult to focus since it dissipates near the body surface. Therefore, LFUS applications are mainly suitable for superficial tumors such as skin, head, neck, or gynecological cancers [19].

Therapies have been developed using high-intensity focused US (HIFU), as an alternative to surgery, for the destruction of tumor tissue at the US focal point [32]. In addition, systems are available to control the HIFU application in real time by magnetic resonance imaging (MRI) or US imaging guidance [33]. During prolonged HIFU-treatment of large tumors, the surrounding normal tissues may be harmed. The use of nanosensitizers with high heating efficiency in combination with HIFU can overcome this problem. The interaction of both leads to more selective and higher tissue damage at the tumor site, while the power and duration of US can be reduced [34]. LIFU (low-intensity focused ultrasound) application is, unlike HIFU, not correlated with much energy accumulation at the focal zone. LIFU is applied in preclinical cancer therapy studies, mainly to improve drug control and release from smart micro-/nanoparticles, and to induce US-related cellular effects [35].

### 2.3. Biological US-Effects

**Figure 2:**
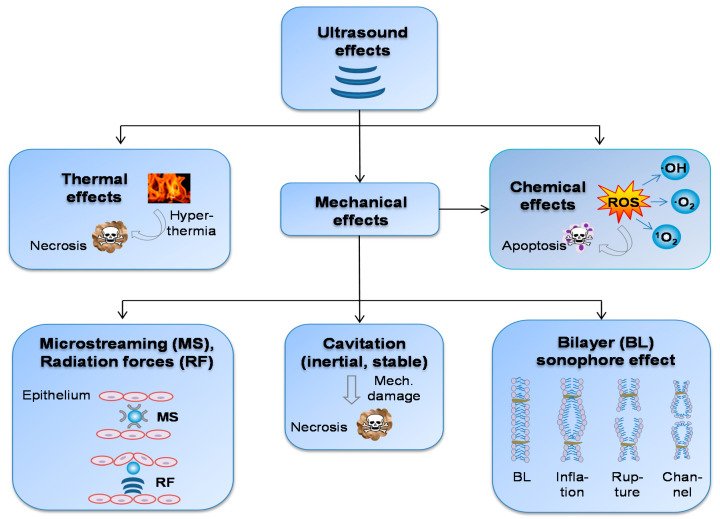
Overview of physico-chemical US effects leading to biological effects on the tissue/cell level.

Several biological effects induced by US are exploited for treatment and drug delivery applications. They can be divided into two main groups: thermal effects, mechanical effects (occurring simultaneously), and, in their succession, also chemical effects (Figure 2).

#### 2.3.1. Temperature Impact on Biological Effects

A propagating US wave carries energy, which can partly be absorbed by the tissue in form of heat. This leads to a temperature increase in the respective area [31]. The thermal index (TI) provides a quantitative estimation of a potential temperature elevation of tissues exposed to US waves and is defined as the ratio of the acoustic power attenuated by the tissue (W_p_) and the power required to increase the tissue temperature by 1 °C (W_deg_) [14]. The increase in tissue temperature is not only dependent on the initial pressure, but also on the frequency (high initial pressure and high frequency lead to high temperature increase). If the temperature increase is high enough, the tissue shows burn and necrosis due to the denaturation of proteins [26]. The sensitivity to temperature varies between different tissues and depends on their protein composition and on the exposure time. It has been observed that 43 °C is a transition temperature. Below this temperature (mild hyperthermia), cell death is reduced. Temperatures above 43 °C (strong hyperthermia) lead to faster protein denaturation and necrosis [36]. Strong hyperthermia is applied for tumor tissue ablation, using HIFU with a local temperature increase up to 50–80 °C [37]. Due to the dilatation of blood vessels and a higher permeability of vessel walls induced at mild hyperthermia (temperature range from 37–43 °C), the blood flow in tissues increases [38]. In addition, mild hyperthermia makes tumor tissue more susceptible to irradiation and chemotherapeutics [39]. Furthermore, mild hyperthermia induces drug release from thermo-sensitive nanocarriers [40] (see below).

#### 2.3.2. Mechanical Impact on Biological Effects

The mechanical effects caused by US can be grouped into non-cavitational effects and acoustic cavitation [41]. Acoustic radiation forces comprise mechanical US effects such as radiation pressure, radiation torque and acoustic streaming, which are not associated with cavitation [42,43] and are able to increase the convective transport of drugs into a region of interest [44]. In addition, shear forces that occur upon US exposure at the fluid/tissue interface [45] can also widen the intracellular space between endothelial cells and may thus enhance nanoparticle penetration into the adjacent tissue [45,46]. The ultrasonic phenomenon of cavitation is widely employed in biomedicine and is based on the formation and/or oscillation of gas bubbles in a fluid [47,48]. The gas bubbles can be of endogenous or exogenous origin. Endogenous bubbles are small gaseous pockets naturally occurring within tissues. Exogenous gas bubbles include synthetic gas bubbles externally administered [49].

**Figure 3:**
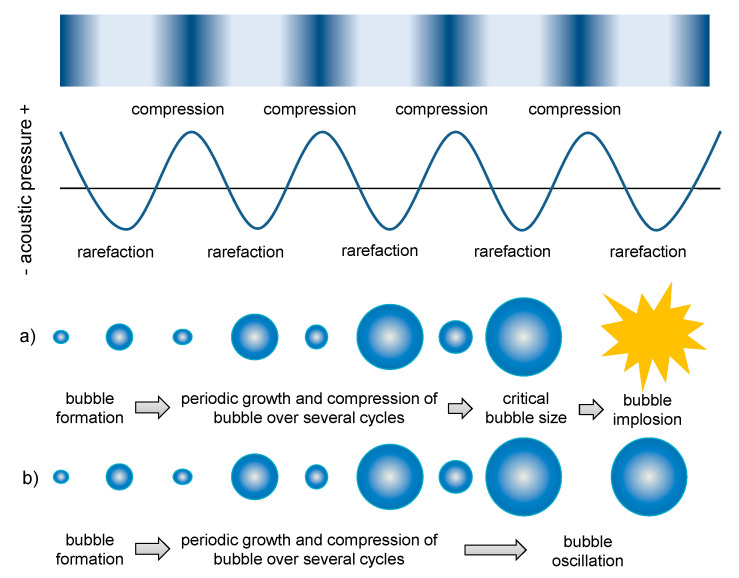
Schematic depiction of (**a**) inertial and (**b**) stable cavitation (reprinted with permission from [51], SPringer, 2018.).

The interaction of the nano-/micrometric sized bubbles with US waves, which are a succession of waves with a negative and positive peak in pressure, leads to oscillation of bubbles in size (growth in the depression phase and shrinking during the compression phase) [50]. Cavitation occurs in two different types (stable and inertial cavitation, see also Figure 3), depending on several parameters including US frequency, pressure, surface tension and available space. Stable (sustained, non-inertial) cavitation is characterized by oscillation of bubbles in the same frequency as the applied US frequency around their resonance size (equilibrium) [26]. This oscillation creates a fluid flow (microstreaming) with velocities and shear forces, which can be strong enough to break particles or to permeabilize cells of the surrounding tissue [27]. By contrast, at higher peaks with negative pressures exceeding a threshold, the bubbles rapidly grow and finally collapse. This is referred to as inertial cavitation.

This effect is exploited by the UTMD (ultrasound targeted microbubble destruction) technique for enhanced drug delivery [52]. The implosion of the gas-filled bubbles generates shockwaves, reaching very high pressures and a temperature increase at the cavitation spot up to 5000 K.

This represents extreme conditions in the local environment. These special conditions can lead to emission of light bursts (sonoluminescence), tissue disruption (exploited for sonoporation and sonophoresis to enhance drug transport), and formation of reactive oxygen species (ROS) due to pyrolysis of water molecules, which induces chemical reactions (sonochemistry) [27,53]. The intracellular production of ROS induced by US also influences the cell membrane permeabilization, which can be exploited for the delivery of therapeutic agents. In addition, ROS can induce cell death in tumors due to their high toxicity and their function as signaling molecules for apoptosis in cancer [54].

The probability of the occurrence of inertial cavitation in a fluid exposed to US depends on the magnitude of energy delivered and is characterized by the mechanical index (MI). The MI is defined as the ratio between the negative pressure peak and the square root of the US frequency applied. The pressure threshold necessary to achieve acoustic cavitation can be decreased by usage of cavitation nuclei (formed from nanoparticles encapsulating a heavy gas core) [55]. This is advantageous for biomedical applications, since, in this case, pressures are sufficient that can be safely applied without damaging healthy tissue [48]. Besides lipid composed micro-/nanobubbles (bubble liposomes) [56], phase-changing nanodroplets [57] and polymer particles are commonly used as cavitation agents as well [58,59].

Effects of US on the cellular level also include (a) an increase in intracellular calcium transients, (b) plasma membrane potential changes, and (c) alterations in cell membrane fluidity. The spontaneous increase in intracellular Ca^2+^ due to US effects on the cell membrane is a reversible process [60] and plays an important role for cell restoration after sonoporation [61]. In addition, it stimulates the endocytosis in cells [62]. There is a direct relationship between increased intracellular Ca^2+^ levels, hyperpolarization of cell membranes and enhanced uptake of micromolecules by pino- or endocytosis [63]. It has been observed that changes in membrane potential associated with sonoporation can result either in hyperpolarization or depolarization, depending on the extent of sonoporation and the applied US intensity [64].

#### 2.3.3. Bilayer Sonophore Effect

An additional, non-thermal, non-cavitational US effect was proposed in [65], called the bilayer sonophore effect. The principle is based on the direct interaction of US pressure waves with bilayer membranes due to their fluctuation between positive (compression) and negative (rarefaction) values. The space between the bilayers increases during the rarefaction phase and decreases during the compression phase. This leads to a short-term disruption of the membrane integrity, rendering substances able to cross the plasma membrane. Consequently, the direct or indirect interaction of US with cell membranes altering the membrane fluidity affects the cell function. Since cellular membranes absorb energy from US radiation, it has been found that the cell morphology can be reversibly altered due to compression or stretching of the cytoskeleton [66]. The transient alterations in cell morphology also influence the lipid bilayers of the cell membrane regarding deformation and thickness. In consequence, this effect leads to the stimulation of gated ion channels of the cell membrane and thus to a change in the intracellular electrolyte distribution [67]. In addition, a reduction of membrane fluidity can occur due to lipid peroxidation induced by US [68].

#### 2.3.4. Alteration of Biodistribution

Due to the physical characteristics of US and its biological effects on the tissue/cellular level, US application can also influence and alter the biodistribution of nanoparticles. In this context, the nanoparticle administration route (local/topic or systemic) and the target tissue/cells play important roles as well [69].

## 3. Concepts in Ultrasound-Triggered Drug Delivery

Based on the biological and physical US effects and the properties of nanocarriers used, different concepts in US-mediated drug delivery can be pursued. These cover the exploration of direct US effects on particles, biological US effects at the intended nanoparticle site of action, and combinations thereof. More specifically, they include acoustic cluster therapy (ACT), hyperthermia, ultrasound targeted microbubble destruction (UTMD) and sonoporation, sonoprinting, sonodynamic therapy (SDT), and acoustic droplet vaporization (ADV). Details are given in Table 1.

Furthermore, there are concepts which are not only restricted to US-mediated drug delivery but used also in other contexts of nanoparticle drug delivery (see Table 2).

## 4. Role of Nanocarrier Properties for Ultrasound-Triggered Drug Delivery

The aim of US-triggered drug delivery is to enhance the drug concentration selectively at the target site. Nanocarrier properties play an important role in this regard [14], whereby their importance is not only restricted to US-mediated drug delivery but also applies to other NP-based drug delivery modes. Physicochemical characteristics such as size, geometry, elasticity, surface features, and composition of nanoparticles have a major impact on their bioavailability, pharmacokinetics, and biocompatibility [9,85], and can be modified to maximize the treatment efficiency of a specific tumor.

The size of nanoparticles is important for their transport in the bloodstream and subsequent delivery to tumor tissue. Due to the leakiness of the tumor vasculature, smaller nanoparticles accumulate more easily in the tumor tissue than larger ones, but they can also extravasate into normal tissue, causing adverse side effects. Larger nanoparticles are not able to extravasate as easily, which provides some selectivity but also reduces the extravasation into tumor tissue and makes their distribution in the bloodstream highly variable [86].

The shape of nanocarriers determines their fluid dynamics and thus the uptake into tumor tissue as well. Typically, nanoparticles have a spherical shape, but recent research revealed that non-spherical nanoparticles tend to drift towards the walls of blood vessels, thus increasing their contact and potential binding to endothelial cells [87].

The stability and blood distribution of nanoparticles is also dependent on their charge. Positively charged nanoparticles are more rapidly eliminated from the bloodstream than negatively charged ones. If the charge of nanoparticles is neutral or slightly negative, the circulation half-life in the blood stream is considerably increased compared with their cationic counterparts [88]. Positively charged nanoparticles were previously shown to most effectively target tumor vessels, but after extravasation a switch to a neutral charge resulted in a faster diffusion of the nanoparticles into the tumor tissue [89].

A further aspect relevant with regard to prolonged blood circulation is the elasticity of nanoparticles. By filtration through the pancreas and liver, rigid nanoparticles are more easily removed from the bloodstream compared with elastic materials, which remain for longer in the blood circulation [88].

Coating of nanoparticles with polyethylene glycol (PEG), which is a hydrophilic and non-ionic polymer, may increase the solubility of nanoparticles and is able to prevent their elimination from the bloodstream by the reticulo-endothelial system (RES). The reduction in nanoparticle clearance increases the probability of reaching the tumor tissue [90], but a disadvantage of PEGylation is that the cellular nanoparticle uptake is significantly decreased. This effect is known as PEGylation dilemma [91]. The surface modification of nanoparticles with ligands may also lead to a prolonged blood circulation, while promoting cellular uptake into tumor tissue [92].

The physico-chemical characteristics of nanoparticles can also be the reason for some practical limitations, which have to be overcome before being able to use nanoparticles for drug delivery. For instance, their small size can cause particle aggregation, making their physical handling difficult and rendering nanoparticle suspensions instable. Furthermore, nanoparticles can show particle growth, unpredictable gelation tendency, limited drug loading and burst release [93].

Ideal properties of nanoparticles intended for use in cancer treatment (and diagnosis) include (a) increased bioavailability and stability against rapid biodegradation, (b) sufficient circulation time and efficient action, (c) ability to pass body barriers, (d) targeted and controlled drug release, (e) minimal toxic side effects, (f) multi-purpose usage (e.g., for diagnostic and therapy) to minimize numbers of applications, and (g) high drug loading capacity [94,95].

## 5. Materials Used for Nano-/Microparticle Development

A wide variety of organic and inorganic materials are used for designing different US-responsive drug delivery nanocarriers such as proteins, liposomes, polymers, polymer-lipid hybrids, phase change materials, and others [92]. Polymeric nanoparticles ranging in the size from 10 to 10,000 nm are of colloidal character and consist of natural polymers such as proteins (e.g., albumin, gelatin, collagen) or polysaccharides (e.g., alginates, chitosan, dextran), or can be fabricated by using synthetic polymers [96], which are usually self-assembled and can be engineered towards various degrees of complexity [97]. It is possible to customize several key properties of these nanoparticles such as molecular weight, biocompatibility, biodegradability, and hydrophobicity [92]. Polymers used for this type of nanoparticles include polylactic acid (PLA), poly-d,-l-lactic glycolic acid (PLGA), polyethylenimine (PEI), polystyrene, and polyalkyl acrylate [98].

PLA and PLGA have been widely used to encapsulate anticancer drugs since these materials can be hydrolyzed in the human body under formation of biocompatible and metabolizable moieties (lactic acid and glycolic acid) that are eliminated from the human body through the citric acid cycle [99]. Thus, unwanted accumulation in tissues can be avoided. Moreover, PLA, and PLGA have already been approved for medical use and are suitable for introducing various targeting moieties to their surface, providing them with tumor-targeting capability. PEGylation of these nanocarriers may prolong their blood circulation time [100]. Furthermore, these colloidal carriers allow the delivery of drugs at high concentrations to a desired location [101], including the co-delivery of therapeutic and imaging agents. This makes them suitable also for usage in the theranostic field [102].

## 6. Drug Release Mechanisms and Nano-/Microparticle Types

Major classes of drug vehicles activatable by US include nano-/microbubbles, micelles, liposomes, mesoporous silica nanoparticles (MSN) as well as perfluorocarbon containing nanodroplets. The drug is dissolved, entrapped, encapsulated or attached to/into a nanoparticle matrix. Depending on the method of preparation, nanoparticles, nanospheres, nanocapsules, nanocups, or others can be obtained [59,93].

The interaction of US and drug-carrying nanoparticles, designed to be responsive to thermal or mechanical stimuli, leads to their disruption and subsequent release of the therapeutic payload. Therefore, it is possible by US application to selectively trigger drug release from nanocarriers within the desired area. In addition, drug delivery systems can be fabricated that are sensitive to multiple (US) stimuli [103]. Multiple-triggered systems allow a stepwise drug release and improve the spatiotemporal control of dosage [69].

### 6.1. Thermal Drug Release and Liposomes

Thermal drug release is based on US-induced temperature increase in the sonicated zone due to the absorption of acoustic energy. This is usually associated with moderate intensities (several W/cm^2^), high duty cycles (up to 100%), moderate pressures (100′s of kPa to MPa) and potentially long treatment times (several seconds to 30 min) using dedicated focused ultrasound (FUS) transducers, with the long treatment time being a primary drawback. To avoid unspecific heating damage, carriers are often designed to deliver their payload at temperatures just a few degrees above physiological temperature (42–43 °C) [104].

Temperature-sensitive liposomes (TSL) are the most common thermally responsive nanocarriers described in the literature [105]. Liposomes are concentric spherical structures in the size range of 20 nm–1 µm, typically formed as membrane bilayers of phospholipids and, in some cases, cholesterol [10,106], separated by aqueous compartments. The aqueous compartments can be used for encapsulating hydrophilic drugs, whereas lipophilic drugs can be incorporated into the membrane [105,106]. For example, ThermoDox^®^ (Celsion Corporation, Lawrenceville, NJ, USA) is a heat-activated drug delivery system already tested in clinical trials that facilitates targeted delivery of a cytotoxic drug (DOX) to tumors at temperatures exceeding 40 °C [107].

Beyond liposomes sensitive to temperature, non-thermosensitive liposomes exist as well, which can be further classified into conventional, fusogenic, pH-sensitive, cationic, long circulatory, and immunoliposomes [105,108]. The similarity of liposome bilayers to cell membranes may contribute to their biocompatibility and cell uptake, by enabling fusogenic liposomes to fuse with cell membranes or with membranes of intracellular compartments. Other liposomes may be endocytosed without fusion.

The drug diffusion through the liposomal lipid bilayer is lower in the case of hydrophilic drug molecules, while hydrophobic or amphiphilic drugs may not be efficiently retained in liposomes. This can potentially lead to toxic effects in normal tissues. Polymeric shielding around the lipid bilayer can prevent outward diffusion of hydrophobic and amphiphilic drug molecules. However, depending on the polymer used, this may also impair the fusion with the target cell and the release of the payload, thus requiring an additional mechanism of drug release. On the other hand, polymeric coatings can alter the surface charge of liposomes and may have further benefits, such as increasing circulation time and targeting efficiency; thus, in some cases, improving the bioavailability of the encapsulated drug [109].

Furthermore, drug-loaded liposomes can be attached to microbubbles, which then release their payload at the desired site upon applying the UTMD method [52]. For example, Gao et al. [110] prepared a microbubble-liposome complex carrying two different cytotoxic drugs (irinotecan loaded in microbubbles and oxaliplatin loaded in liposomes) followed by applying the UTMD method, which represents a mode of drug release that relies on mechanical US stimuli (see Table 3).

### 6.2. Mechanical- and Multiple-Triggered Drug Release

Micro-/nanobubbles, liquid perfluorocarbon droplets, micelles or mesoporous silica nanoparticles (MSN) are drug carriers exploiting mechanical (e.g., cavitation, shear forces) or multiple-triggered (US-) effects for drug release, respectively [104].

#### 6.2.1. Microbubbles

Microbubbles (MBs) are already FDA approved with regard to human safety (e.g., SonoVue^®^ sulphur hexafluoride microbubbles). They were originally developed as ultrasound contrast agents (UCAs) [111,112] and are extensively investigated also for US-mediated drug/gene delivery applications [113]. Due to their size (typically 1–8 µm), microbubbles must be considered as intravascular agents that do not extravasate. They consist of a protein (albumin), lipid, surfactant or biocompatible polymer shell (thickness: 2–500 nm) surrounding a gaseous core (e.g., perfluorocarbon (PFC) gas and air mixture). The composition of the shell determines the bubble stiffness which impacts their resistance to rupture in the US pressure field and their susceptibility for recognition and clearance by the reticuloendothelial system [114].

Phospholipids are among the most-applied surfactants for surface coating of bubbles. They have a hydrophilic head and two hydrophobic tails, which spontaneously form a monolayer around the gas core. A wide variety of modifications can be implemented on the amphiphilic lipid surface, including antibody surface decoration, the incorporation of drug molecules inside the hydrophobic shell or their attachment to the shell through covalent or non-covalent (electrostatic) binding. In addition, drug-loaded nanoparticles can be encapsulated into or attached to the surface of microbubbles [115]. Another technique of drug loading is based on the inclusion of a drug-containing oil-phase within the microbubble [116].

Generally, sonication of drug-loaded microbubbles has the advantage of simultaneously inducing both local drug release and cell membrane permeabilization (e.g., by the UTMD method; see above). In addition, the drug-loaded microbubble can be visualized by low-intensity US which can be exploited for image guided drug delivery [117]. Furthermore, it is possible to use microbubbles for enhanced drug delivery by applying the UTMD technique and co-injection of drug loaded nanoparticles [118,119]. Disadvantages of using microbubbles for drug delivery include their limited capacity for loading therapeutic agents, their short circulation time, and large micrometer size, making it difficult to achieve efficient drug concentrations in the tumor area [120]. In addition, the injection of microbubbles may cause adverse side effects, such as high osmotic pressure and blood vessel dilation [79,121].

#### 6.2.2. Nanobubbles

A possible way to overcome these limitations may rely on converting microbubbles to nanobubbles (size: 5–500 nm) or developing acoustic phase shift nanodroplets which are able to extravasate more easily through endothelial gaps and accumulate in the tumor tissue [122]. The nanoscale associated problems of low echogenicity and instability of nanobubbles could be overcome by modification of nanobubbles (NBs) with pluronic acid. A comparison of pluronic acid modified NBs with microbubbles revealed that the modified NBs exhibited equal or better echogenicity and longer circulation time, and that NBs synthesized with pluronic surfactant were more stable [123].

Volatile liquid acoustic phase shift nanodroplets stabilized as emulsions are explored as an alternative to conventional exogenous microbubbles using the method of acoustic droplet vaporization (ADV) [124]. These nanodroplets are typically composed of a liquid PFC core and coated materials (typically lipid) which are stable in the blood stream [125]. Combined thermal and mechanical US stress can be used to induce the vaporization of the gas-precursors. When insonified, the liquid core rapidly converts into gas, leading to the disruption of the droplets and to rapid expansion of their content [104]. Nanodroplets also represent a class of multifunctional stimuli-responsive nanocarriers: they combine the properties of passive-targeted drug carriers, US imaging contrast agents, and US-responsive drug delivery systems [125]. Cluster formation of microdroplets (oil droplets) with microbubbles is exploited for a related technology, termed acoustic cluster therapy (ACT) [74]. Liu et al. [126] prepared and investigated phase-changeable folate-targeted perfluoropentane nanodroplets loaded with 10-hydroxycamptothecin (HCPT) and superparamagnetic Fe_3_O_4_ for multimodal tumor imaging and targeted therapy in mice. LIFU was employed for activating ADV. The combination of LIFU and the described nanodroplets were considered to be an ideal modality for tumor targeted theranostics, since a distinct tumor growth inhibition could be achieved as well as an improvement of MR/PA imaging (see also Table 3).

#### 6.2.3. Micelles

Micelles are self-assembled colloidal structures from polymer molecules (unimers), which have reached a critical concentration in an aqueous solution (the so-called “critical micelle concentration”, CMC). In addition, the self-assembly depends on a certain threshold temperature (critical micellar temperature, CMT) [126]. Micelles are typically in the size range of 10–100 nm and are mostly synthesized from amphiphilic di-block or tri-block copolymers. In contrast to liposomes, micelles are composed of monolayers, with a hydrophobic core (serving as drug reservoir) and a polar surface area (hydrophilic shell). Hydrophilic shells form steric barriers, preventing micelle aggregation, and promote the solubility of micelles in an aqueous environment [127]. Usually, micelles are spherically shaped, but other morphologies (e.g., rods, lamellae) can be designed as well, depending on the characteristics of the constituent blocks of the polymer and the temperature [128].

Polyethylene oxide (PEO) and polyethylene glycol (PEG) are hydrophilic blocks most commonly used for micelle formation. They have identical monomer subunits (-CH2-CH2-O), but the end groups differ depending on the synthesis procedure [126]. The PEG blocks are able to prevent micelle aggregation as well as micelle opsonization. Thus, micelles are less recognized by the RES, which enhances the plasma residence time [129].

The choice of hydrophobic blocks, such as poly-L-amino acids and biodegradable polyesters, is mainly dependent on the drug compatibility with the core [126]. Poloxamers are nonionic triblock copolymers composed of a central hydrophobic chain of polyoxypropylene flanked by two hydrophilic chains of polyoxyethylene (Pluronic^®^), which are widely employed for the formation of micelles used in acoustically activated drug delivery [44]. Due to the chemical flexibility of the micelle structure, a variety of modifications can be implemented in order to develop “tailor made” drug carriers, such as the formation of cross-links between the polymer chains to improve stability against premature dissociation under physiological conditions [126]. These crosslinks, however, should be biodegradable (with gradual micelle dissociation into unimers) to prevent micelle accumulation in the body. Furthermore, ligands such as antibodies, oligosaccharides, peptides, or others can be attached to the hydrophilic shell, for targeted drug delivery.

Modifications can also aim at making the micelles responsive to different stimuli (US, heat, light, lower pH in tumor environment) for triggered drug release. Thermo-responsive micelles can be combined with US heating, but the mechanical effects of US can also be exploited for micelle-based drug delivery [127]. Nelson et al. [130] applied stabilized doxorubicin loaded Pluronic^®^ P-105 micelles (Plurogel) for colon cancer treatment in rats, in combination with low-intensity focused ultrasound. A significant reduction in tumor size compared with non-insonated controls was obtained. In this case, three synergistic US effects likely occurred: (i) augmentation of micelle extravasation, (ii) DOX release, and (iii) increase in intracellular drug uptake (see also Table 3).

#### 6.2.4. Mesoporous Silica Nanoparticles (MSNs)

Solid nanoparticles are another group of nano-entities applicable for US mediated drug delivery. They are characterized by spherical structures with a solid core [106]. One of the most promising nanoparticles of this type for drug delivery are mesoporous silica nanoparticles (MSNs). MSNs are inorganic nanosystems providing a high drug-loading capacity due to the material inherent pores, which considerably increase the surface area. For preventing premature release of therapeutic cargo through diffusion, it is necessary to block these pores by molecules acting as pore caps. The caps will then only detach from the MSNs upon application of certain stimuli such as US (mechanical and/or thermal stress), thus triggering cargo release [131]. Besides high drug-loading capacity, MSNs provide further advantages such as biocompatibility and physicochemical robustness. In addition, MSNs can be easily equipped with different moieties and thus acquire more specific functions (e.g., targeted therapy [132], physiological stabilization [133], long circulation times [134]), thus making them a versatile tool not only for drug delivery, but also for imaging or theranostic applications [135]. However, the lack of their biodegradability is one of the main biosafety issues since the remnants of MSNs would deposit in organs which may cause systemic toxicity and organ damage due to difficulties in excretion [136]. Therefore, great efforts have been undertaken to optimize the biodegradation kinetics of MSNs, such as surface modification and organic-inorganic hybridization [137]. Li et al. [138], developed an US reversible response MSN nanocarrier modified with sodium alginate and carboxyl-calcium coordination bonds in the modified layer, which could block the mesopores of MSN and effectively prevent the cargo from being prematurely released prior to stimulation. The coordination bonds could be destroyed by applying LIFU (20 kHz) or HIFU (1.1 MHz), leading to a rapid and significant cargo release, and were recovered when the US was turned off, resulting in an instant cargo release stopping. These hybrid MSN-based nanoparticles had excellent, reversible ultrasound on–off responsiveness and could be of great interest for on-demand drug delivery applications.

### 6.3. Natural Nanocarriers: Exosomes

Besides chemically synthesized nanocarriers, exosomes have recently emerged as promising “natural drug delivery carriers” [139]. Exosomes are phospholipid bilayer nanovesicles (size: 40–120 nm) secreted by most cell types, including B cells, T cells, dendritic cells, macrophages, neurons, glial cells, tumor cell lines, and stem cells. These extracellular vesicles are able to deliver various cargoes (proteins, lipids, DNA, RNA) between cells within the organism and play a major role in distant cell-cell communication. Due to their small size and decoration with cell surface molecules, they are also able to overcome various biological barriers and have an inherent targeting capacity preventing off-target effects [140]. Comparable to liposomes, the phospholipid bilayer of exosomes surrounds a hydrophilic core which enables their loading with polar drugs [141]. These natural properties of exosomes makes them highly interesting as carriers for therapeutic payloads [140]. In the context of exosomes, US is relevant in several respects. Firstly, the application of US has been demonstrated as useful for promoting the biosynthesis of exosomes in order to increase their yield for drug delivery [142]. Additionally, US treatment can facilitate drug loading into exosomes [143] and enhance their drug delivery efficiency [139,144].

## 7. Applications In Vivo

As described above, cancer treatment using US-mediated drug delivery is a complex process, with treatment efficiencies depending on several factors such as the type of nanocarrier and the US parameters used, the type of drug, the tumor type and size, drug dosage, treatment regimen, and others. An overview of several US-responsive nanocarriers in combination with different US applications and their therapeutic outcome in vivo is presented in Table 3. The treatment efficiency is expressed as percentage of tumor volume reduction (TVR) or volume inhibition rate (VIR). Efficiencies are given for NP applications with and without US, compared with empty vehicle administration. In some cases, US application has been described as an alternative for non-US treatment. In most studies, considerable tumor inhibition rates (>50%) were achieved, in some cases even upon NP treatment without US application. However, as expected, the treatment efficiency including US was always superior or at least comparable to that of non-US treatment.

Obviously, it is more desirable to not only to achieve a tumor growth inhibition, but a reduction in tumor volume (preferably up to 100%). Three studies listed in Table 3 were able to obtain these results in different ways. Kheirolomoom et al. [145] achieved a complete regression of murine NDL breast cancer in mice by applying temperature sensitive liposomes (TSLs) in combination with US-mediated hyperthermia. The liposomes contained a pH-sensitive copper-doxorubicin (CuDOX) complex that dissociated in low pH environments with free DOX release, thus representing a hierarchical drug delivery system. All mice treated with CuDOX-LTSLs combined with US survived, and tumor was not detectable 8 months post treatment.

A HIFU approach combined with liposomal cerasomes; i.e., organic-inorganic vesicular nanohybrids, here containing a polyorganosiloxane surface was used by Liang et al. [146]. They achieved a 96% TVR of a human breast cancer xenograft in mice. The temperature-sensitive nanohybrid cerasomes were fabricated by introducing LTSLs lipid components into cerasomes, thus obtaining nanocarriers with prolonged blood circulation time compared with conventional LTSLs and tunable release characteristics. A complete and stable tumor (human breast cancer xenografts) regression in all mice treated was also obtained by Snipstadt et al. [147], when applying NP stabilized MBs combined with FUS. The MBs were formed by self-assembly of NPs into a shell. Different US intensities tested in this study revealed that a mean acoustic pressure of MI 0.5 led to enhanced tumor uptake without tissue damage. At a higher MI of 1, however, tissue damage was observed while lower acoustic pressures (MI 0.1 and 0.25) did not enhance the tumor uptake.

The same human breast cancer xenograft mouse model as above was also employed in cancer treatment studies by Zhu et al. [148], Xu et al. [149], and Kim et al. [150] pursuing different concepts of US-mediated drug delivery. Zhu et al. developed phase-transformation lipid NPs functionalized with the peptide tLyP-1, exhibiting targeting and penetrating efficiency. Synergistic effects of ADV and UTMD were exploited under LIFU treatment. In addition, US imaging was enhanced due to microbubble formation. Mesoporous silica nanoparticles (MSN) loaded with doxorubicin (DOX) and the sonosensitizer chlorin e6 (Ce6) were applied by Xu et al. [149] in sonodynamic therapy to treat cancer while Kim et al. used membrane fusogenic liposomes (MFLs) loaded with docetaxel in combination with FUS-induced microbubble cavitation. Significant tumor volume inhibition rates (>50%) could be achieved with all three methods applied, albeit no tumor regression, such as in the studies of Liang et al. [146] and Snipstadt et al. [147], were found (see also Table 3).

**Table 3:** Therapeutic outcome of in vivo studies using different modalities in US-mediated drug delivery.

Particles: Composition	Drug/Dose [mg/kg]	US Method/Principle of Action	US Parameters/HT Protocol	Animal/ Tumor	TV [mm³] at TrS (d = 0)	Results after TIm/TrS at Day	TVR [%]/ VIR [%]		Remarks/ Special Features	Ref.
							(+) US	(-) US		
MBs: 1. oxygen and PTX loaded PFC-MB (OPLMBs)	PTX/ 20	Non-FUS/ UTMD	Freq.: 300 kHz, Intens.: 1 W/cm^2^, DC: 50 %, Duration: 10 s	Mice (f)/s.c. human ovarian cancer (SKOV3)	70–100	22 (TIm); 8 (TrS)	95 VIR	67 VIR	Simultaneous enrichment of oxygenation and selective delivery of drugs at the tumor site; efficacy: OPLMB > PLMB	[151]
2. PTX loaded PFC-MB (PLMBs)							81 VIR	62 VIR		
MBs: DSPC|DPPC|DPPA (PFP)	DTX/ N/A	Non-FUS/ UTMD	Freq.: 300 KHz, Intens.: 2 W/cm^2^	Rabbit (f, m)/ rabbit liver tumor (VX2)	~450	22 (TIm); 8 (TrS)	31 VIR	9 VIR	Slight TV suppression (< 50% VIR)	[152]
MBs: DPPC|DPPA|DPPE-PEG2k (PFP)	HCPT/ 4	LIFU/ UTMD	Freq.: 1 MHz, Intens.: 2 W/cm^2^	Mice/s.c. murine hepatoma (H22)	~50	15 (TIm); 8 (TrS)	71 VIR	48 VIR	MB formulation with high loading capacity for HCPT	[153]
MBs: DPPC|DPPG|DPPE-PEG2k (C_3_F_8_)	DOX/ N/A	LIFU/ UTMD	Freq.: 1.3 MHz; MI: 1.6	Rat (m)/s.c. murine pancreas cancer (DSL6A)	N/A	14 (TrS)	70 VIR	27 VIR	MB formulation with high loading capacity for DOX	[154]
LPs (MFL) + MBs: DMPC|DOTAP|DSPE-MPEG2k + SonoVue^®^	DTX/ 2	FUS/ UTMD	Freq.: 1.1 MHz, Power: 20 W, PRF: 40 Hz; DC: 5%	Mice (f) /s.c. human breast cancer (MDA-MB-231)	~150	28 (TrS)	(+) MBs 55 VIR	(-) MBs 33 VIR	MFLs did fuse well onto cell membrane for intracellular drug delivery; MB + FUS led to sonoporation of vascular cells and to enhanced EPR effect	[150]
LPs (TSLs): DPPC|DSPE-PEG2k|MPPC-CuDOX	DOX/ 6	FUS/HT	US pulses consisted of 100-cycle bursts at 1.54 MHz, PRF: 100 Hz–5kHz, HT: 42 °C, 5 min prior to NP injection and 20 min after	Mice/murine NDL breast cancer	≥ 30	28 (TrS)	100 TVR		pH-sensitive complex between DOX and copper (CuDOX); remains associated at neutral pH, but dissociates and releases free DOX in lower-pH environments	[145]
LPs (HTSCs): CFL|DPPC|MSPC|DSPE-PEG2k	HDOX/ 5	HIFU	DC: 30%, Voltage: 190 mV for 5 min twice: immediately and 24 h after injection, 42 °C	Mice (f)/s.c. human breast cancer (MDA-MB-231)	~106	16 (TrS)	96 TVR	70 TVR	HTSCs with high physiological stability and tunable release characteristics, by introducing LTSLs lipid components into cerasomes	[146]
LP-MB complex: 1. LPs.: DPPC|DSPE-PEG2k-biotin|CHOL; 2. MBs: DSPC|DSPE-PEG2000| DSPE-PEG2000-Biotins (C3F8); 3. Avidin-bridge	PTX/ N/A	FUS	Burst length: 10 ms, DC: 1%, PRF: 1 Hz, Duration: 10 min	Mice (f)/s.c. murine breast cancer (4T1)	65–270	21 (TIm); 11 (TrS)	71 VIR	29 VIR	Increased apoptosis and reduced angiogenesis achieved	[155]
LPs + MBs: 1. LPs: Doxil^®^; 2. MBs: DPPA|DPPC|DPPE-PEG2k|Glyc. (C_3_F_8_)	DOX/ 10	LIFU/ UTMD	Freq.: 1.1 MHz, Intens.: 2.06 W/cm^2^, MI: 0.48	Mice (f)/s.c. murine hepatoma (H22)	N/A	20 (TIm); 16 (TrS)	80 VIR	62 VIR	Effective and safe treatment combination of Doxil^®^ and UTMD	[156]
1. LPs (Doxil-like) + MBs: HSPC|CHOL|MPEG2000-DSPE + SonoVue^TM^	DOX/ 6	LIFU/ UTMD	Freq.: 1 MHz, MI: 0.8, pulses with 10 000 cycles	Mice (f)/s.c. human prostatic cancer (PC3)	100–200	28 (TrS)	(+) MBs 58 VIR	(-) MBs 17 VIR	PEG cleavage of coated LPs by MMP enzymes led to increased intracellular uptake compared to NES- LPs, but VIR: Doxil-like > ES > NES	[157]
2. LPs (enzyme sensitive, ES) + MBs: POPC|CHOL|PCL + SonoVue^TM^							(+) MBs 39 VIR	(-) MBs 6 VIR		
3. LPs (non enzyme sensitive, NES) + MBs: POPC|CHOL|MPEG2000-CHOL + SonoVue^TM^							(+) MBs 21 VIR	(-) MBs 12 VIR		
LP-MB complex 1. LPs (OX): CHOL|DPPC|DSPE-PEG2k-biotin; 2. MBs (IR): DBPC| DSPE-PEG2k, DSPE-PEG2k-biotin (PFB); 3. Avidin bridge	OX/ ~1; IR/ ~ 5	LIUS/ UTMD	Freq.: 1 MHz, Intens.: 3.5 W/cm^2^, DC: 30%, PRF: 100 Hz, PNP: 0.48 Mpa, MI: 0.48	Mice (f)/s.c. human pancreatic cancer (BxPC-3)	~100	14 (TrS)	90 VIR	44 VIR	Dual drug loading (OX loaded LPs, IR loaded MBs)	[110]
LPs: Caelyx^®^	DOX/ 1	LFUS	Freq.: 20 kHz, continuous wave, Intens.: > 3.16 W/m²	Mice/s.c. human colon cancer (WiDr)	N/A	21 (TrS)	56 VIR	47 VIR	Non-hyperthermic US treatment shows significant effect on tumor growth; occurrence of synergistic effects between US and drugs at lower concentrations	[158]
	DOX/ 6						72 VIR	72 VIR		
Micelles: Plurogel (Pluronic P105 stabilized with NNDEA)	5-FU/ 100	LFUS	Freq.: 20 kHz, continuous wave, Intens.: >3.16 W/m²	Mice/s.c. human colon cancer (WiDr)	N/A	21 (TrS)	33 VIR	16 VIR		
	5-FU/ 200						49 VIR	49 VIR		
Micelles: Plurogel (Pluronic P105 stabilized with NNDEA)	DOX/ 2.7	LFUS	Freq.: 70 kHz, Intens.: 2 W/cm^2^ Power train: 1:10 pulse (0.2 s on, 1.8 s off)	Rat/s.c. rat colon cancer (DHD/K12/TRb)	N/A	~49 (TrS)	96 VIR, partly TVR	76 VIR	Probably 3 synergistic US effects occurred: 1. micelle extravasation ↑, 2. DOX release, 3. intracellular drug uptake ↑	[130]
Micelles: Pluronic^®^ P-105 with PEG2k-DSPE	DOX/ 3	Non-FUS	Freq.: 1 MHz, Intens.: 3.4 W/cm^2^, DC: 50 %, Duration: 30 s	Mice (f)/s.c. human ovarian cancer (A2780)	75–125	21 (TrS)	90 VIR	80 VIR	Stabilization of Pluronic^®^ P-105 micelles with PEG2000-DSPE; high drug-loading capacity; no enhancement of micelle extravasation by US, but intracellular drug uptake ↑	[159]
Synthetic polymer NP-MB complex: 1. NP: PLGA; 2. MB: DPPC|DSPE-PEG-NH2|PLL|Glyc (C_3_F_8_)	DOX/ N/A	LIFU/ UTMD	Freq.: 1 MHz, Intens.: 1.2 w/cm^2^, DC: 50%, Duration: 60 s	Rabbit/ rabbit liver tumor (VX2)	N/A	26 (TIm); 11 (TrS)	57 VIR	N/A	Targeted destruction of MBs by LIFU was superior in comparison to non-FUS	[160]
							Non- FUS: 43 VIR			
MBs stabilized by polymeric NP: PEG-PEBCA (C_3_F_8_)	CTX/ 10	FUS	Burst length: 10 ms, DC: 2.5%, PRF: 0.5 Hz, Duration: 2 min. MI: 0.5	Mice (f)/s.c. human breast cancer (MDA-MB-231)	20–30	43 (TIm); 22 (TrS)	100 TVR	83 VIR	Lower acoustic pressures (MI of 0.1 or 0.25) did not enhance tumor uptake of NPs, tissue damage observed at MI of 1	[147]
Synthetic polymer NPs + MBs: MPEG-PLGA-PLL–anti CA19-9 + SonoVue^®^	PTX/ 2	LIFU/ UTMD	Freq.: 1 MHz, Intens.: 2 W/cm^2^, DC: 20 %, Duration: 2 min	Mice (m)/s.c. human pancreatic adeno-carcinoma (Capan-1)	50–100	31 (TIm); 21 (TrS)	(+) MBs 91 VIR	(-) MBs 83 VIR	Ab-mediated active targeting	[161]
Phase-transformation lipid NPs: DPPG|DPPC|CHOL|DSPE-PEG3.4k-tLyP-1 (PFP)	HCPT/ N/A	LIFU/ ADV, UTMD	Freq.: 1 MHz, Intens.: 3.2 W/cm^2^, Duration: 1 s with a 1 s pause for a total of 3 min	Mice (f)/s.c. human breast cancer (MDA-MB-231)	100	14 (TrS)	67 VIR	40 VIR	tLyP-1 peptide with targeting and penetrating efficiency; synergistic effects of ADV and UTMD, enhanced imaging through MB formation	[148]
Phase-transformation lipid NPs: PFP|DPPC| DC-CHOL| DSPE-CPPs|HA	HCPT/ 4	LIFU/ ADV, UTMD	Intens.: 3.2 W/cm^2^, DC: 50 %, Duration: 2 min	Mice/s.c. human hepatoma (SMMC-7721)	~512	31 (TIm); 11 (TrS)	95 VIR	79 VIR	CPPs/HA with targeting and penetrating efficiency; synergistic effects of ADV and UTMD, enhanced imaging through MB formation	[162]
Phase-changeable NDs: 1. FA-modified lipid shell (PL, CHOL) 2. Fe_3_O_4_ 3. PFP core	HCPT/ 4	LIFU/ ADV, UTMD	Intens.: 3.2 W/cm², pulsed-wave mode	Mice (f)/s.c. human ovarian cancer (SKOV3)	400–500 (d = 2)	14 (TrS)	74 VIR	52 VIR	Ligand (FA)-mediated active targeting, synergistic effects of ADV and UTMD, multimodal tumor imaging (MRI, PAI)	[163]
MD-MB cluster: 1. MDs: PFMCP| DSPC 2. MBs: Sonazoid^®^	PTX/ 15	FUS/ ACT	Activation: Freq.: 1.5 MHz, PRF: 26.1 Hz DC: 0.18 %, MI: 0.44, Duration: 45 s; Treatment: Freq.: 0.3 MHz, PRF: 100 Hz DC: 7.28 %, MI: 0.1, Duration: 300 s	Mice (m)/s.c. human pancreatic ductal adenocarcinoma (PDAC) (MIA PaCa-2^luc^)	50–80 (AV: ~53)	45 (TIm); 31 (TrS)	ACT-PTX: 86 VIR, partly TVR	PTX: 72 VIR	Two frequencies required: treatment efficiency also dependent on activation efficiency	[164]
MSN encapsuled in MBs: 1. NPs: MSN-folate 2. MBs: DPPC|DPPE|Glyc. (C_3_F_8_)	TAN/ 8	LIUS/ UTMD	Freq.: 1 MHz, Intens.: 2 W/cm^2^	Mice (m)/s.c. murine hepatoma (H22)	~150 (d = 3)	8 (TrS)	64 VIR	43 VIR	High drug loading capacity, multitargeting capability	[165]
MSN: MSN-Ce6	DOX/ 3	FUS/SDT+NP	4 W/cm^2^	Mice (f)/s.c. human breast cancer (MDA-MB-231)	N/A	9 (TrS)	MSN-DOX-Ce6: 88 VIR	N/A	Synergistic effects of SDT and DDS (may be enhanced by introducing targeting molecules); high drug-loading properties	[149]
							DOX+ Ce6: 62 VIR			

## 8. Toxicological and Biosafety Considerations

Even though the development of (smart) nanoparticle drug delivery systems offers several advantages compared with conventional chemotherapy, there are major concerns regarding their toxicity [13]. It is thus necessary to address these potential toxicity issues for human health prior to the translation of promising technologies into therapeutic clinical applications. Due to the unique properties of nanomaterials, conventional drug toxicity assays may be insufficient or inadequate for the full assessment of nanoparticle toxicity. In addition, there is no standard list of required tests. Therefore, it remains difficult to evaluate the toxicity of nanomaterials and the development of validated advanced complementary assays, indicating the need of standard criteria for toxicity assessment [166,167].

The immune system responds to foreign stimuli including nanoparticles and thus serves as primary defense against foreign invasion [168]. Exposure of the immune system to nano-objects can lead to inflammation and allergic/autoimmune reactions. Antigenic characteristics of nano-objects, inflammatory effects and their ability to activate the complement system determine the extent and type of immunological reactions at which immune response can then be either stimulated or suppressed [85]. Nano-immuno interactions are therefore important to be considered in the process of the development of such drug delivery systems.

Recently, the safety of repeated exposure to PEG has been questioned, since repeated administration of PEGylated drug formulations was shown to lead to hypersensitivity reactions and increased clearance rates due to activation of the complement system and antibody formation against PEG [169]. This is of significant concern since the use of PEG in drugs, cosmetics and others has climbed almost exponentially since its discovery. Still, these side effects may be tolerated in the case of patients with life-threatening diseases [170].

In the context of nano-immuno interactions, special attention has to be devoted to testing nanomaterials for possible bacterial endotoxin contaminations that may occur especially when the nanomaterials are produced under non-sterile conditions or in the presence of water [85]. Since endotoxins are potent immune stimulants that can elicit a cytokine storm, they can confound the results of toxicity and efficacy studies [171].

Nanoparticles may cause several cytotoxic effects that are unwanted in non-tumor tissue. These include oxidation via ROS formation and other free radicals altering the membrane flexibility leading to cell death, damage of cell membranes by perforating them, disturbance of intracellular transport and cell division by damaging cytoskeleton components, induction of DNA damages enhancing mutagenesis, damage of mitochondria leading to cell energy imbalance, interference with lysosome formation and triggering apoptosis, structural changes in membrane proteins disturbing the transport of substances and activation of the synthesis of inflammatory mediators leading to the disturbance of cell metabolism as well as tissue and organ metabolism [172]. Since the toxicity of nanoparticles strongly depends on their physicochemical characteristics, a comprehensive material characterization is a critical requirement for each nanotoxicological study and will lead to a better understanding on how different nanoparticle properties affect their biological response [173].

Physicochemical nanoparticle properties such as size, shape, surface charge, surface structure, agglomeration and aggregation, hydrophilicity, stability, chemical composition as well as the presence or absence of a shell and of active groups on the surface affect the ADME (absorption, distribution, metabolism, and excretion/elimination) behavior and may cause adverse biological responses [85,172]. Size and surface charge seem to be the most important parameters in toxicity assessment of nanocarriers. Toxicity and nanocarrier size are inversely related; the smaller the nanoparticle size, the higher the toxicity and vice versa [174,175]. While smaller nanoparticles (10–15 nm) have a widespread biodistribution, larger nanoparticles tend to accumulate in organs (liver and spleen) of the mononuclear phagocyte system (MPS) [85]. Due to their small size, nanoparticles will not only penetrate easily through epithelial and endothelial barriers into the lymph and blood to be transported to different organs and tissues [176,177], but are also able to enter cells and cell organelles (e.g., mitochondria and nuclei). This may drastically affect cell metabolism and cause DNA lesions, mutations, and cell death [178]. Several studies revealed that nanoparticle shape is important regarding toxic effects as well. For example, spherical nanoparticles can be more easily engulfed by endocytosis than nanotubes and nanofibers [179].

The surface charge of nanoparticles largely determines their interaction with biological systems. It is known that nanocarriers with positive charges show greater toxicity compared with those with negative or neutral charges. This can be explained by the ability of cationic nanoparticles to easily enter cells due to their interaction with negatively charged cell membrane glycoproteins [172]. In addition, cationic nanoparticles show an enhanced capacity for opsonization, i.e., nanomaterials in biological environments are subject to adsorption of proteins facilitating phagocytosis, including antibodies and complement components [180]. The adsorbed proteins, referred to as the protein “corona”, will affect the surface properties of nanoparticles. Coronas are dual-layered systems composed of an inner core of strongly bound proteins (“hard corona”) and an outer layer of more loosely bound molecules (“soft corona”) that undergo adsorption and desorption more rapidly [181]. The protein corona is not static and can change depending on the direct environment of the nanocarrier. Furthermore, other biomolecules such as lipids can also adhere to the nanomaterial surface [85]. The structure and composition of the protein corona is influenced by factors such as the synthetic identity of the nanomaterial (inherent physicochemical properties) [182], nature of the physiological environment (e.g., blood, interstitial fluid, cell cytoplasm), and duration of exposure [183]. The protein corona formation alters the size and interfacial composition of a nanomaterial, which thus obtains a biological identity that is different from its synthetic identity. Therefore, physiological responses including signaling, kinetics, transport, accumulation and toxicity are influenced by this acquired biological identity [85].

The toxicity of nanoparticles is also determined by their chemical composition and biodegradation. For example, liposomes are considered to be biocompatible systems due to their phospholipid and cholesterol structure [184], whereas toxic synthesis components, or by-products such as heavy metals, should be avoided [185]. In addition, the biodegradation process of nanoparticle material should not lead to toxic products. Biodegradable nanopolymers such as PLA and PLGA can be completely broken down and are often used for nanocarrier preparation to minimize toxicity [99]. Furthermore, many types of nanoparticles are not easily recognized by the protective systems of the body. This may decrease the rate of their degradation and may lead to considerable accumulation of nanoparticles in tissues, even to highly toxic or lethal concentrations [172]. In this context, the nanoparticle doses applied and the time intervals between the applications play an important role as well. When the clearance capacity of the body is exceeded, nanoparticle accumulation will occur. For example, high or repeated nano-object dosages injected into the bloodstream can overwhelm the phagocytic cells in the liver and spleen, leading to a redistribution of the nanoparticles to other organs [85]. The excretion of micro-/nanoparticles should thus be controlled in order to reduce the accumulation risk of foreign materials within the body [186].

Since nanocarriers for cancer treatment may contain highly cytotoxic drugs, it is important that the nanocarrier exhibits a certain degree of stability to prevent premature drug release which would affect healthy tissues. A nanoparticle shell may not only be able to increase the stability of the nanocarrier, but also its solubility in water and biological fluids by decreasing its aggregation capacity. Beyond improving the nanoparticle biocompatibility, the shell may provide nanoparticles with the capacity for selective interaction with different types of cells and biological molecules. This may influence their pharmacokinetics and change their distribution and accumulation patterns in the body. Therefore, a shell around nanocarriers plays a major role in (the reduction of) nanocarrier toxicity [187]. Finally, the route of administration also determines the toxicity of nanoparticles since their biodistribution and toxicokinetics are altered depending on the exposure route [85].

As described above, unintentional accumulation of nanocarriers within healthy tissue prior to delivery of the drug to the tumor site should be avoided. Microbubbles are considered to be too large to readily extravasate into healthy tissue; over time, however, the microbubbles including their drug load will have a chance to spread further and accumulate in liver and spleen when these organs perform their natural clearance functions. As these microbubbles degrade, the drugs will be released and possibly reach toxic levels [12]. At present, long-term biocompatibility evaluations are often lacking and toxic effects of nanocarriers in specific organs have not been fully elucidated. The paucity of solid biosafety data is thus considered as a major reason for hindering the further clinical translation of such micro-/nanoplatforms [186].

In the case of US-triggered drug delivery systems, the application of US is an additional important biosafety issue. As described above, the extent and severity of thermal and mechanical US effects depends on several US parameters such as frequency, focusing, pulse repetition frequency, pulse duration, exposure time, and intensity, as well as on the attenuation coefficient and acoustic impedance of biological tissues. This is relevant also in this context since, as a result, the thermal and mechanical effects advantageous for cancer treatment might also affect healthy tissues leading to adverse biological effects [188]. For example, during HIFU treatment, the very high level of US energy around the focus inevitably damages healthy tissues, leading to severe side effects such as transient pain, skin burns and nerve injury. In addition, US application can lead to unwanted cavitation effects in the presence of residual air bubbles. Gas-containing organs such as lungs and bowels are thus not suitable for US-based treatment. For limiting the risks of thermal or mechanical injuries caused by US, appropriate indices (thermal index, mechanical index) have been introduced (see Section 2). These indices are thus helpful for the development of effective and safe modalities for tumor-specific imaging and therapy under consideration of the biological effects of US [14].

Taken together, the development of efficient US-triggered drug delivery micro-/nanoplatforms with tolerable side effects is a complex process influenced by multiple parameters that need to be balanced against each other. A feature considered as advantageous in a given therapeutic setting may lead to adverse effects in another situation. Consequently, the comprehensive understanding of the biological interactions that take place inside an animal or human system, beginning from nanoparticle administration until its final fate, is essential for the development of nanoscale drug delivery systems [9].

## 9. Conclusions and Outlook

Ultrasound-mediated drug delivery is a versatile tool providing several advantages compared with conventional chemotherapy for cancer treatment, such as improving the efficiency and reducing the toxicity of a given drug therapy. Different nanoparticle-based US-mediated drug delivery systems and appropriate concepts have already been developed. They may need some further optimization in the light of issues described above, but their translation into the clinics can be well anticipated.

Obviously, the potential toxicity of nanoparticles and their components needs to be sufficiently tested and addressed. At present, the availability of biocompatibility data, especially for long-term biosafety evaluation and toxicity to specific organs, and of specific guidelines may still be considered as bottlenecks for the clinical application of nanoparticle-based drug delivery systems. A multidisciplinary approach is most promising for addressing the complexity of developing US-responsive drug delivery systems. On the other hand, the possibility of (i) additively or synergistically combining the effects of physical and pharmacological intervention, (ii) using intelligent and stimulus-responsive systems for spatially confined drug delivery, and (iii) exploring the advantages of nanotechnology in this regard is particularly intriguing. Nanoparticle systems are already in clinical use for improving drug pharmacokinetics or for extending the spectrum of possible drugs (e.g., towards RNA molecules). Thus, the use of ultrasound-responsive nanocarriers for cancer treatment is a clearly realistic scenario.

## Figures and Tables

**Table 1:** Concepts in US-triggered drug delivery.

Concept	Principle	Additional Information
Hyperthermia	- induces drug release from specially designed thermo-sensitive nanoparticles (e.g., Thermodox^®^) [40] - leads to increased blood flow and fenestration in heated tumor tissue, resulting in improved nanoparticle accumulation in tumors [70,71]	- see also Section 2.3.1
Ultrasound targeted microbubble destruction (UTMD) and sonoporation	- combines low frequency moderate power US with microbubbles for triggering cavitation, thus obtaining the sonoporation effect [72]	- Sonoporation: biophysical process that is based on stable or inertial acoustic cavitation of microbubbles; used for enhancing the permeability of plasma membranes through the generation of short-lived pores - see also Section 2.3.1
Sonoprinting	- based on US application to nanoparticle-loaded microbubbles, which leads to a direct deposition of nanoparticles along with parts of the bubble shell onto cell membranes, followed by cell internalization after several hours	- novel mechanism of using microbubbles for drug delivery, as recently proposed by Cock et al. [73].
Acoustic cluster therapy (ACT^®^)	- comprises i.v. administration of free-flowing clusters of negatively charged microbubbles and positively charged microdroplets (oil droplets) - co-administration of drugs or loading of microdroplets with lipophilic therapeutic agent - initiation of vaporization process of the oil droplet by activating the clusters with US - production of large gas bubbles by inwards diffusion of blood gases (20–30 µm) and transient occlusion of blood flow (~5–10 min) - drug release from microdroplets due to cluster activation into the local blood compartment - induction of biomechanical effects by further US application, leading to increased vascular permeability and locally enhanced extravasation of components from the vascular compartment (e.g., released or co-administered drugs) [74]	- immediate drug wash out is avoided due to the transient occlusion of the vessel, and the drug is kept locally at high concentrations for a certain period of time
Sonodynamic therapy (SDT)	- three key elements necessary: low-intensity US, special agents known as sonosensitizers and molecular oxygen - principle relies on the accumulation of sonosensitizers in the tumor tissue and their activation by the action of ultrasonic cavitation - interaction of the induced sonosensitizer with the surrounding oxygen molecules will lead to the generation of reactive oxygen species (ROS) and eventually to the irreversible destruction of the targeted tumor tissue [75]	- approach complementary to photodynamic therapy; here, however, US instead of light is used as the external stimulus [76]
Acoustic droplet vaporization (ADV)	- technique employs volatile liquid acoustic phase shift nanodroplets that are typically composed of a PFC core and lipid coating - penetration of small nanodroplets into the extravascular stroma tissue of tumors followed by US exposition - PFC droplets inside the coating then undergo a phase transition from liquid into an expanding gas bubble - major effect of ADV is inertial cavitation (see 2.3.1) [77] - expansion of the droplets during the transition process to form gas bubbles leads to disruption of the lipid coating and thus a rapid release of the drug content [78] - in addition, inertial cavitation effect of ADV can also cause physical disruption at the tumor site [79]	- first introduced by Kripfgans et al. [80]

**Table 2:** Concepts not only restricted to US-triggered drug delivery.

Concept	Principle	Additional Information
Surface functionalization of nanocarriers	- modification of NP surfaces to achieve desired NP properties and behavior, such as stimulus-responsiveness, targeting, stability and others [13]	- e.g., nanoparticles can be PEGylated for escaping the RES and thus for increasing the blood circulation time. Further possible surface modifications include the binding of antibodies or ligands to enhance target-specific drug delivery [10]
Co-delivery	- simultaneous transport of different agents such as therapeutic drugs and imaging agents [80], two chemotherapeutic drugs, oligonucleotides and chemotherapeutics [81] or chemotherapeutics and anticancer metals [82]	- offers promising strategies for increasing therapeutic efficacies
Multiple triggered systems	- systems sensitive to multiple stimuli - can be applied as hierarchical platforms, which are based on changeable particle sizes, switchable surface charges and activatable surface ligands - potentially enhancing both, tumor tissue accumulation/retention and cellular internalization of nanocarriers - stepwise drug release possible [69,83]	- e.g., exposure of a moiety on the NP surface that induces uptake after a certain environmental condition is present (e.g., heat, low pH, enzymes)
Theranostic approach	- Theranostics: combined term derived from the words ‘diagnosis’ and ‘therapy’, meaning that diagnostic imaging and therapeutic treatment can be carried out using a single multifunctional nanomaterial	- development of image-guided drug delivery systems is possible (e.g., US or MRI imaging combined with US-mediated drug delivery) [84]

## Data Availability

Not applicable.

## Abbreviations

5-FU	5-fluorouracil
Ab	Antibody
ACT	Acoustic cluster therapy
ADME	Absorption, distribution, metabolism and excretion/elimination
ADV	Acoustic droplet vaporization
AV	Average; C3F8, Perfluoropropane; Ce6, chlorin e6 (sonosensitizer)
BL	Bilayer
CFL	Cerasome-forming lipid
CHOL	Cholesterol
CPP	Cell penetrating peptide
CPT	Camptothecin
CTX	Cabacitaxel
DC	Duty cycle (effective ultrasound emission rate)
DC-CHOL	3-(*N*-(*N*’,*N*’-Dimethylaminoethane) carbamoyl) cholesterol
DMPC	1,2-dimyristoyl-sn-glycero-3-phosphocholine
DOTAP	1,2-dioleoyl-3-trimethylammonium-propane
DPPA	1,2-dipalmitoyl-sn-glycero-3-phosphatidic acid
DPPC	1,2-dipalmitoyl-sn-glycero-3-phosphocholine
DPPE	1,2-dipalmitoyl-sn-glycero-3-phosphoethanolamine
DTX	Docetaxel
ECM	Extracellular matrix
EPR	Enhanced permeation and retention
ES	Enzyme sensitive
FA	Folic acid
FUS	Focused ultrasound
Glyc	Glycerol
HA	Hyaluronic acid
HCPT	10-hydroxycampthothecin
HDOX	Hydrophilic doxorubicin hydrochloride
HIFU	High-intensity focused US
HT	Hyperthermia
HTSC	HIFU and temperature-sensitive cerasome
IR	Irinotecan
ISO	International Organization for Standardization
LFUS	Low frequency ultrasound
LIFU	Low-intensity focused US
LIUS	Low-intensity ultrasound
LP	Liposome
LTSL	Low temperature sensitive liposomes
MB	Microbubble
MD	Microdroplet
MFL	Membrane fusogenic liposome
MI	Mechanical index
MMP	Matrix metalloprotease
MPEG2k	Methoxy polyethylene glycol 2000
MPPC	1-palmitoyl-2-hydroxy-sn-glycero-3-phosphocholine
MPS	Mononuclear phagocyte system
MRI	Magnetic resonance imaging
MS	Microstreaming
MSN	Mesoporous silica nanoparticle
MSPC	Monostearoylphosphatidylcholine
NDL	Neu deletion
NES	Non enzyme sensitive
NNDEA	N,N-diethylacrylamide
NP	Nanoparticle
OPLMB	Oxygen and paclitaxel loaded microbubbles
OX	Oxaliplatin
PAI	Photoacoustic imaging
PCL	PEGylated cleavable lipopeptide
PDAC	Pancreatic ductal adenocarcinoma
PEBCA	Poly-2-ethyl-butyl cyanoacrylate
PEG	Polyethylene glycol
PEG2k	Polyethylene glycol 2000
PEO	Polyethylene oxide
PFB	Perfluorbutane
PFC	Perfluorocarbon
PFMCP	Perfluoromethylcyclopentane
PFP	Perfluoropentane
PL	Phospholipid
PLA	Polylactic acid
PLGA	Poly-d,-l-lactic glycolic acid
PLL	Poly-l-lysine
PLMB	Paclitaxel loaded microbubbles
PNP	Peak-negative pressure
POPC	1-palmitoyl-2-oleoyl-sn-glycero-3-phosphocholine
PPO	Polypropylene oxide
PRF	Pulse repetition frequency
PRP	Pulse repetition period
PTX	Paclitaxel
RES	Reticuloendothelial system
RF	Radiation forces
ROS	Reactive oxygen species
s. c.	Subcutaneous
SDDS	Smart drug delivery systems
SDT	Sonodynamic therapy
TAN	Tanshinone IIA
TI	Thermal index
TIm	Tumor implantation
tLyP-1	Tumor homing-penetrating peptide
TR	Technical report
TS	Technical specification
TrS	Treatment start
TSL	Thermosensitive liposome
TV	Tumor volume
TVR	Tumor volume reduction
UCA	Ultrasound contrast agent
US	Ultrasound
UTMD	Ultrasound targeted microbubble destruction
VIR	Volume inhibition rate
VSSA	Volume specific surface area
